# Molecular Characterization of Three Gonadotropin Subunits and Their Expression Patterns during Ovarian Maturation in *Cynoglossus semilaevis*

**DOI:** 10.3390/ijms16022767

**Published:** 2015-01-27

**Authors:** Bao Shi, Xuezhou Liu, Yongjiang Xu, Shanshan Wang

**Affiliations:** Qingdao Key Laboratory for Marine Fish Breeding and Biotechnology, Key Laboratory of Sustainable Development of Marine Fisheries, Ministry of Agriculture, Yellow Sea Fisheries Research Institute, Chinese Academy of Fishery Sciences, Qingdao 266071, Shandong, China; E-Mails: shibao@ysfri.ac.cn (B.S.); xuyj@ysfri.ac.cn (Y.X.); 0610wangshan@163.com (S.W.)

**Keywords:** *Cynoglossus semilaevis*, multiple spawner, gonadotropin, cDNA cloning, expression patterns, oocyte maturation

## Abstract

The endocrine regulation of reproduction in a multiple spawning flatfish with an ovary of asynchronous development remains largely unknown. The objectives of this study were to monitor changes in mRNA expression patterns of three gonadotropin hormone (GTH) subunits (FSHβ, LHβ and CGα) and plasma GTH levels during ovarian maturation of half-smooth tongue sole *Cynoglossus semilaevis*. Cloning and sequence analysis revealed that the cDNAs of *FSHβ*, *LHβ* and *CGα* were 541, 670 and 685 bp in length, and encode for peptides of 130, 158 and 127 amino acids, respectively. The number of cysteine residues and potential N-linked glycosylation sites of the flatfish GTHs were conserved among teleosts. However, the primary structure of GTHs in Pleuronectiformes appeared to be highly divergent. The *FSHβ* transcriptional level in the pituitary remained high during the vitellogenic stage while plasma levels of FSH peaked and oocyte development was stimulated. The *LHβ* expression in the pituitary and ovary reached the maximum level during oocyte maturation stages when the plasma levels of LH peaked. The brain *GTHs* were expressed at the different ovarian stages. These results suggested that FSH and LH may simultaneously regulate ovarian development and maturation through the brain-pituitary-ovary axis endocrine system in tongue sole.

## 1. Introduction

The two gonadotropin hormones (GTHs), follicle-stimulating hormone (FSH) and luteinizing hormone (LH), secreted by the pituitary gland, are key modulators of gametogenesis and gonadal steroidogenesis in almost all vertebrates, including fish. These GTHs contain a common glycoprotein α subunit (CGα) that forms a heterodimer with a hormone-specific β subunit (FSHβ or LHβ) [1,2]. The hormones react to specific receptors at the reproductive organs which is an essential requirement for their physiological action [3,4]. It is generally accepted that FSH and LH of fish have complementary functions during reproduction; FSH is mainly involved in the control of puberty and gametogenesis, whereas LH predominates at final gonadal maturation, ovulation or spermiation [2,5,6,7,8].

Different temporal expression patterns of *GTH* subunits suggested the different regulatory mechanisms for endocrine control of oogenesis. Expression patterns of *GTHs* in female fish spawning single batches of eggs, have shown that the *FSHβ* transcription increased during the vitellogenic stage and decreased during final maturation of gametes. However the *LHβ* transcription increased rapidly during final ovarian maturation, correlating the fluctuations of plasma FSH and LH levels in rainbow trout (*Oncorhynchus mykiss*) [9]. On the other hand, GTH regulation mechanisms are not clear for multiple spawner fish, in which different results have been reported depending on the species, the methodologies used or because different oocyte stages coexist in the same ovary [10]. For instance, expression patterns of *GTHs* have been reported during the ovarian cycle of several multiple batch spawners [11,12,13,14,15,16]. It is suggested that both *GTHs* transcription seem to fluctuate in parallel to stimulate the development of asynchronous batches of follicles in multiple spawning fish. Moreover, recently, two clear peaks have been found of plasmatic FSH and LH levels in Nile tilapia (*Oreochromis niloticus*) females in the course of one reproductive cycle, one peak during vitellogenesis and the other during spawning [17]. Some interesting information on the physiological function of GTHs has been revealed through molecular approaches by analyzing gene expression in the tissue of the extrapituitary. In brain of Nile tilapia, the brain-derived GTHs may function as hypophysiotropic hormones and neuromodulators of eproductive behaviors [18]. Moreover, in the ovary of gilthead seabream (*Sparus aurata*), the existence of a gonadotropin-releasing hormone (GnRH)-GTH axis was found. The GTHs may have novel roles in teleost intraovarian communication between oocytes and ovarian follicle cells [19,20]. For better understanding of the mechanism of GTH subunits in the brain-pituitary-ovary endocrine system at different gonadal development stages of multiple spawner fish, further studies should be performed in this field.

Half-smooth tongue sole (*Cynoglossus semilaevis*) is a commercially exploited flatfish that is widely distributed in Chinese coastal waters. This species exhibits a sexually dimorphic growth. In nature, the females grow over three times faster and larger than males. The mature testis is from 1000th to 5000th the volume and weight of the ovary [21]. Moreover, ovarian development in this flatfish is asynchronous with clutches of oocytes undergoing final oocyte maturation, ovulation, and spawning over a period of up to three months. The tongue sole exhibits some degree of reproductive dysfunction under rearing conditions. The major obstacle for its large-scale aquaculture is the control of reproduction in captive stocks, especially of females which often show a low oocyte maturation rate. Thus, it is important to improve our understanding of the reproductive endocrinology of this flatfish and enhance the efficiency of reproduction. Recently, a number of studies have been conducted in this species for potential applications in aquaculture, such as ZW sex chromosome evolution and benthic lifestyle adaptation [22], sexual reversal [23], sex determination [24,25], development and growth [26,27] and reproduction [28,29,30,31]. Up to now, there is no information on the biological functions and mechanisms of GTHs in tongue sole.

In the present study, three *GTHs* cDNA were cloned from tongue sole and the transcript levels quantified in different tissues and in the brain-pituitary-ovary axis endocrine system at the different ovarian development stages, combined with the detection of plasma FSH and LH levels, as well as gonadosomatic indices. The purpose of this study was to elucidate the role of GTH subunits during the ovarian development and maturation of tongue sole by analyzing their mRNA expression patterns and plasma circulating levels of FSH and LH. The results generated in this study provide useful information for artificial propagation and reproduction regulation of the tongue sole.

## 2. Results and Discussion

### 2.1. Gonadal Stages and Changes in Gonadosomatic Index (GSI)

**Figure 1:**
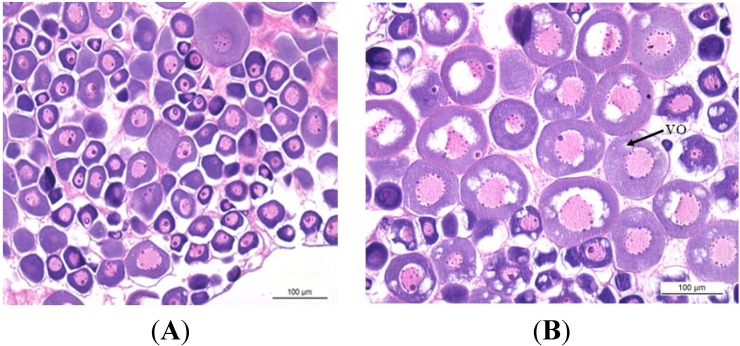

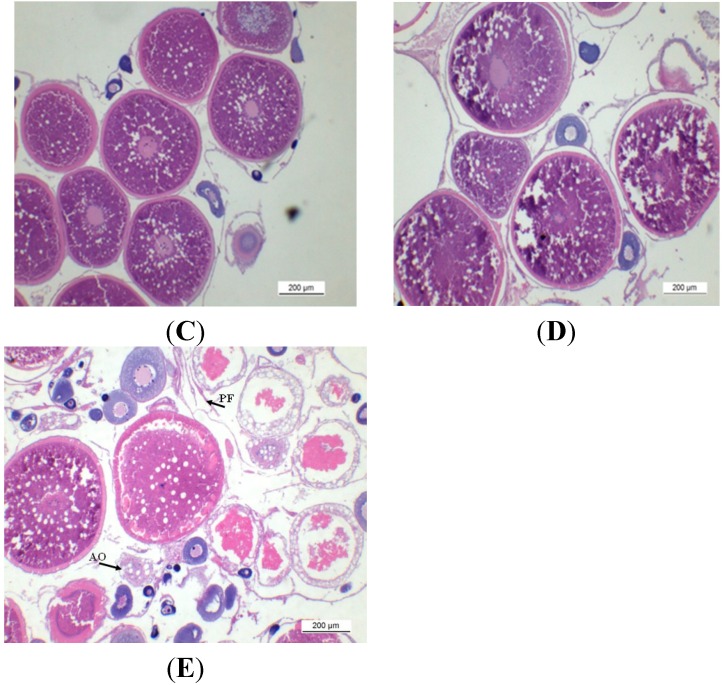
Representative stained sections for each of the five stages of *C. semilaevis* ovarian development. (**A**) previtellogenesis, ovary in stage II, bar = 100 μm; (**B**) vitellogenesis, ovary in stage III, bar = 100 μm; Balck arrow denotes vitellogenic oocyte; (**C**) late vitellogenesis, ovary in stage IV, bar = 200 μm; and (**D**) maturation, ovary in stage V, bar = 200 μm; (**E**) After ovulation, ovary in stage VI, bar = 200 μm; Balck arrows indicate post-ovulatory follicles or atretic oocyte. VO: Vitellogenic oocyte; PF: Post-ovulatory follicles; and AO: Atretic oocyte.

**Figure 2:**
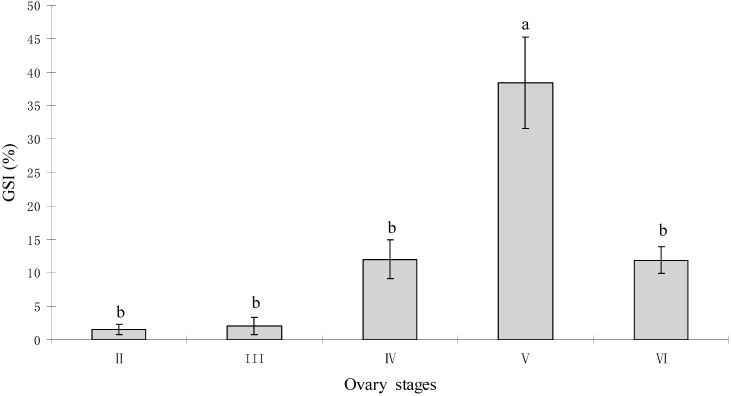
Changes of the Gonadosomatic Index (GSI) values at different ovary stages in *C. semilaevis* (*n* = 4). Each bar represents the mean ± standard error (SE). The values with different letters differ significantly from each other (*p* < 0.05).

Representative sections of ovaries showing the morphological characteristics at different developmental stages of the tongue sole ovary are shown in Figure 1. These flatfish have asynchronous ovarian development with oocytes at different developmental stages at the same time throughout reproduction. The changes in GSI values during this study are shown in Figure 2. The gonadosomatic index (GSI) value was low, when the ovaries were at the previtellogenic (stage II) and beginning of vitellogenic stages (stage III) (Figure 1A,B). The GSI value rapidly increased from the late vitellogenesis stage (stage IV) (Figure 1C) until it reached its highest level at the ovarian maturation stage (stage V) (Figure 1D). A rapid decrease of the GSI value was then observed after ovulation (stage VI), this stage showed mainly atretic oocytes with degenerated late-vitellogenetic oocytes in their ovaries (Figure 1E). Moreover, the GSI value of this stage was higher than the one observed during previtellogenesis.

**Figure 3:**
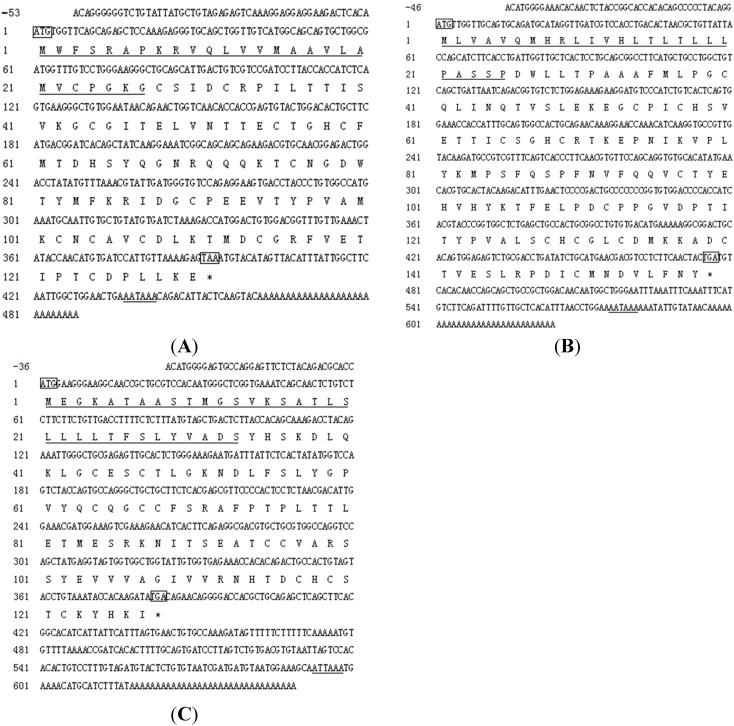
Nucleotide and deduced amino acid sequences of *C. semilaevis* gonadotropin subunits (FSHβ (**A**), LH β (**B**) and CGα (**C**)). Nucleotides (upper sequence) and amino acid (lower sequence) are numbered on the left. Amino acids that comprise the signal sequence are underlined. The start and stop codon are shown by boxes. The stop codon (TAA or TGA) is indicated by asterisks (*). The putative polyadenylation signal (AATAAA or ATTAAA) is underlined.

### 2.2. cDNA Cloning of Tongue Sole Gonadotropin Hormone (GTH) Subunits

The tongue sole *FSHβ* cDNA (GenBank accession number: JQ277933) was 541 bp in length, and consisted of a 5' Untranslated region (UTR) of 53 bp, a coding sequence of 393 bp, and a 3' UTR of 95 bp. A putative polyadenylation signal (AATAAA) was located 24 bp upstream of the poly-A tail (Figure 3A). The predicted mature FSHβ peptide consisted of 103 amino acids preceded by a signal peptide of 27 amino acids. The tongue sole *LHβ* cDNA (GenBank accession number: JQ277934) was 670 bp in length with a 5' UTR of 46 bp, a coding region of 477 bp, and a 3' UTR of 147 bp. A putative polyadenylation signal (AATAAA) was located 17 bp upstream of the poly-A tail (Figure 3B). The putative protein contained 158 amino acids including a signal sequence of 25 amino acids. The tongue sole *CGα* cDNA (GenBank accession number: JQ364953) was 685 bp in length with a 5' UTR of 36 bp, a coding region of 384 bp, and a 3' UTR of 265 bp. A putative polyadenylation signal (ATTAAA) was located 25 bp upstream of the poly-A tail (Figure 3C). The open reading frame (ORF) encoded a mature peptide of 127 amino acids which was preceded by a signal peptide 33 amino acids long.

**Figure 4:**
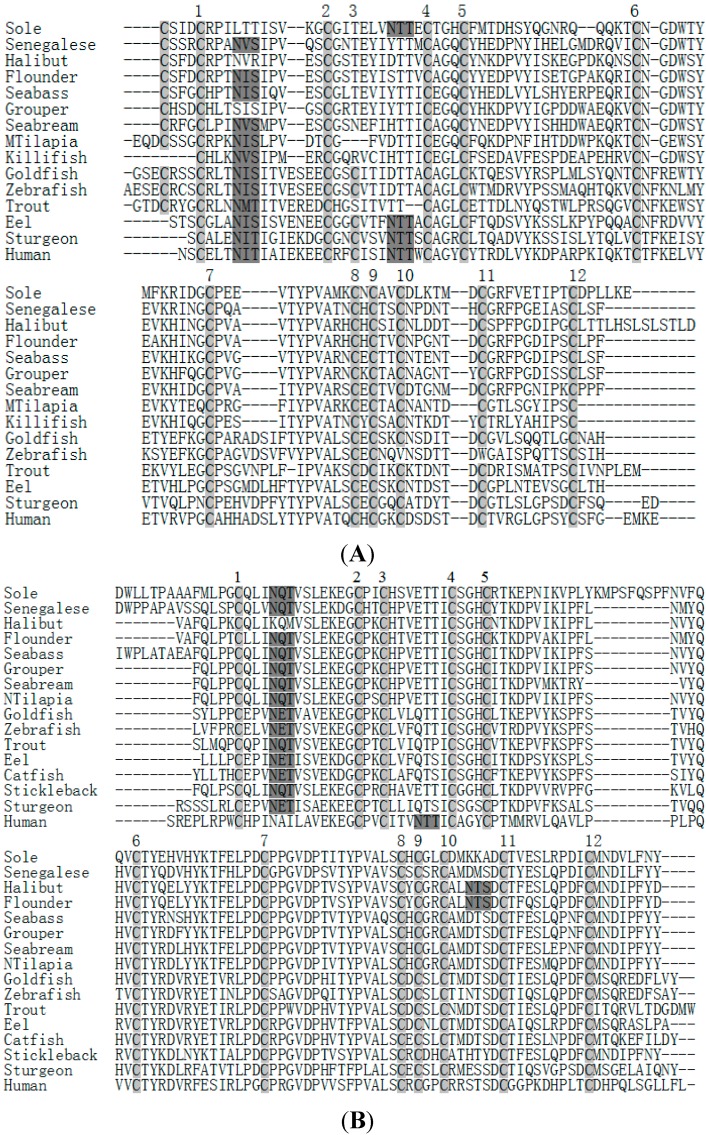

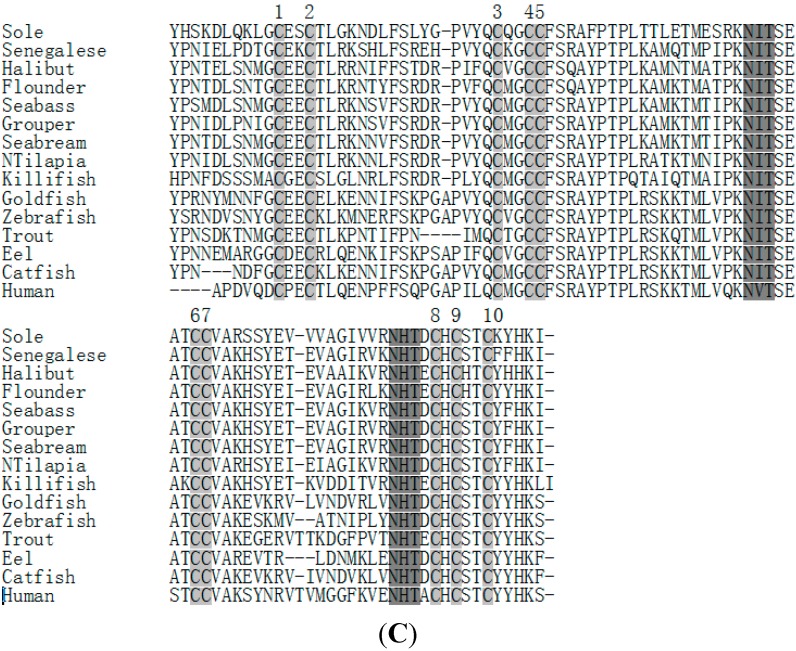
Alignment of amino acid sequences of *C. semilaevis* mature FSHβ (**A**), LHβ (**B**) and CGα (**C**) subunits with those of representative vertebrates. The cysteine residues are marked by gray shadow and numbered from the *N* terminus. The N-linked glycosylation sites are indicated by black shadow. Sole: *Cynoglossus semilaevis*, Senegalese: *Solea senegalensis*, Halibut: *Hippoglossus hippoglossus*, Flounder: *Paralichthys olivaceus*, Seabass: *Dicentrarchus labrax*, Grouper: *Epinephelus coioides*, Seabream: *Pagrus major*, Mtilapia: *Oreochromis mossambicus*, Killifish: *Fundulus heteroclitus*, Goldfish: *Carassius auratus*, Zebrafish: *Danio rerio*, Trout: *Oncorhynchus mykiss*, Eel: *Anguilla anguilla*, Sturgeon: *Acipenser baerii*, Catfish: *Clarias gariepinus*, Stickleback: *Gasterosteus aculeatus*, Ntilapia: *Oreochromis niloticus*, Human: *Homo sapiens.*

### 2.3. Amino Acid Sequence Analysis of Tongue Sole GTH Subunits

The homology analysis based on the amino acid sequence of the tongue sole FSHβ and LHβ mature peptides indicated that they were 22% identical, whereas the tongue sole CGα showed similar identities to FSHβ as to LHβ (9% and 15%, respectively). The mature peptides of tongue sole GTH subunits showed 46% and 49% (FSHβ), 66% and 67% (LHβ) and 67% and 62% (CGα) identity to the homologs of other pleuronectiform teleosts Senegalese sole (*Solea senegalensis*) and Japanese fiounder (*Paralychthys olivaceus*), respectively. However, the same range of sequence identities was observed between tongue sole and perciforms such as European sea bass (*Dicentrarchus labrax*) or orange-spotted grouper (*Epinephelus coioides*). Thus, three mature subunits of tongue sole showed 46% (FSHβ), 72% (LHβ) and 70% (CGα) to the European seabass GTH subunits, and 44%, 74% and 69% identity, respectively, to those of the orange-spotted grouper.

The alignment of mature protein of tongue sole FSHβ, LHβ and CGα with those of other teleosts and human revealed that the Cys residues and putative *N*-glycosylation sites of three GTH subunits were highly conserved. The protein of tongue sole CGα contains 10 Cys residues while FSHβ and LHβ contain 12 Cys residues, respectively. The mature peptide sequences of tongue sole FSHβ and LHβ have one putative *N*-glycosylation site while CGα have two putative *N*-glycosylation sites (Figure 4).

**Figure 5:**
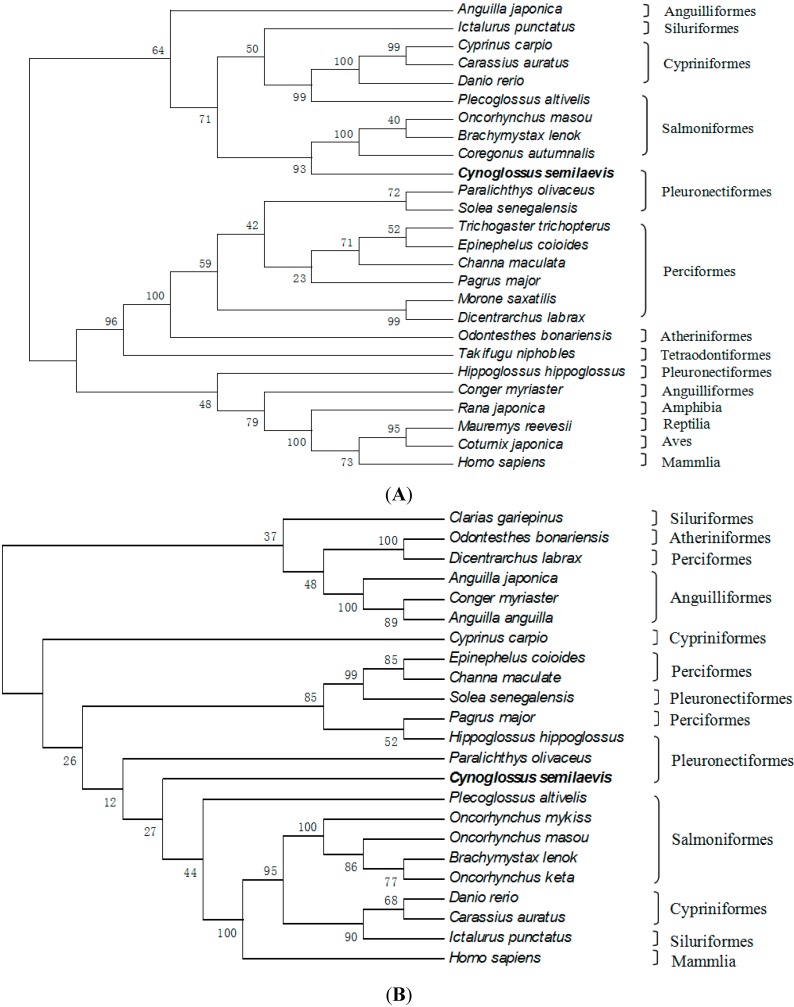

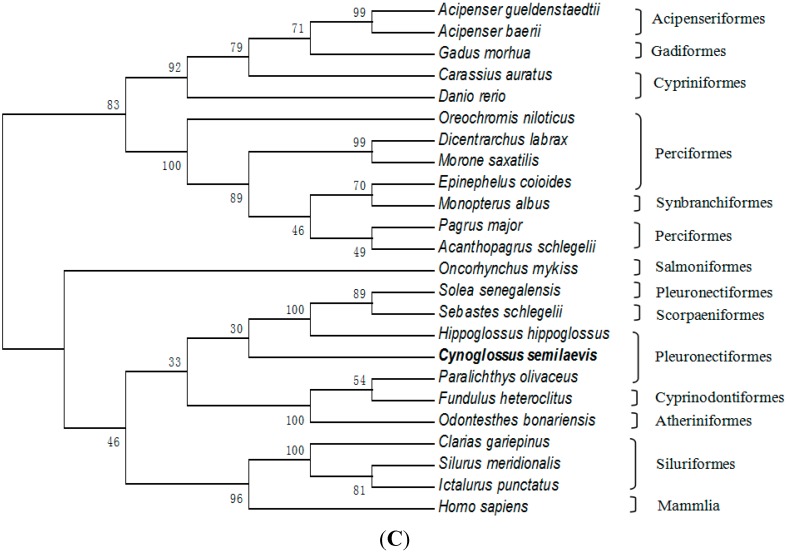
Phylogenetic tree based on the amino acid sequences of FSHβ (**A**), LHβ (**B**) and CGα (**C**) from vertebrate. The tongue sole FSHβ, LHβ and CGα are highlighted by boldface. Bootstrap values (%) are indicated by 1000 replicates.

Interestingly, the sequence of KMPSPQSPF was only found in the mature peptide of LHβ (Figure 4B). One possible explanation may be that the Cynoglossidae fish are highly specialized groups in Pleuronectiformes. Phylogenetic trees constructed for comparative analysis of tongue sole FSHβ, LHβ and CGα with known fish and selected vertebrate sequences are shown in Figure 5. Comparison of amino acid sequences indicated that both tongue sole FSHβ and LHβ were more similar to their orthologs of Percomorphs (Perciforms, Pleuronectiformes; 44%–49% identity) than to Elopomorphs (*Anguilla* sp.; 30% identity) and Ostariophysis (Cyprinids; 27%–31% identity). However, the identity of tongue sole FSHβ and LHβ with those from other Pleuronectiformes (Senegalese sole and Japanese fiounder) was relatively low, 46%–49% (FSHβ) and 66%–67% (LHβ), and similar to those with FSHβ and LHβ from perciforms (44%–46% and 72%–74%, respectively). The amino acid sequence of tongue sole CGα showed the highest identity with the perciform species (69%–70% identity), whereas identity with other teleost groups ranged from 62%–67% for Pleuronectiformes, 58%–60% for Cyprinids and 53%–58% for Salmonids, Anguilliforms and Silurids.

### 2.4. Tissue Distribution of GTH Subunits in Maturing Female Tongue Sole

**Figure 6:**
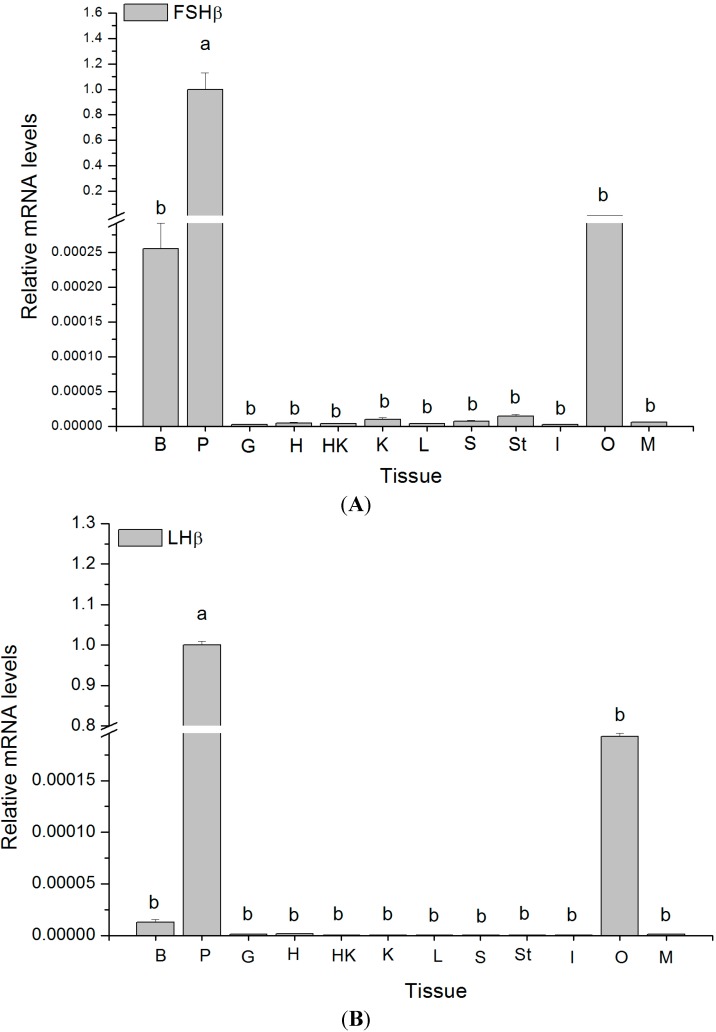

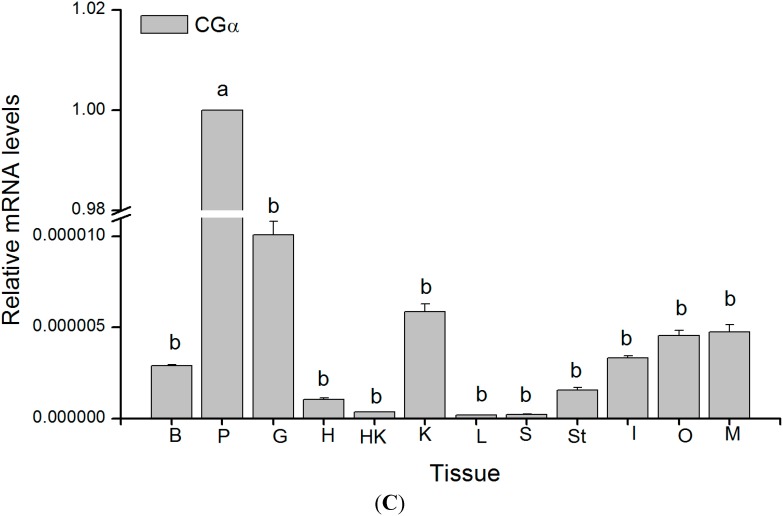
Relative mRNA expression levels in maturing female *C. semilaevis* tissues. The abundance of *FSHβ* (**A**), *LHβ* (**B**) and *CGα* (**C**) transcripts, respectively, was determined by qRT-PCR and normalized to *18S* (*n* = 4). Each bar represents the mean ± SE. Tissues marked by the different letters differing significantly from each other (*p* < 0.05). B: Brain, P: Pituitary, G: Gill, H: Heart, HK: Head kidney, K: Kidney, L: Liver, S: Spleen, St: Stomach, I: Intestine, O: Ovary, M: Muscle.

As expected strong expression of all three *GTH* subunit mRNAs was detected by qRT-PCR in the pituitary. In addition, positive signals were obtained for all three subunits in ovary and brain, although the resulting transcript levels were at least 120-fold lower than in the pituitary (Figure 6). Weak but still positive signals resulting in very low transcript levels of *FSHβ* mRNA were obtained when analyzing kidney, as well as stomach (Figure 6A). Additionally, *LHβ* mRNA was detected in the heart, liver and kidney whereas *CGα* mRNA became positive in the gill, kidney and intestine (Figure 6B,C).

### 2.5. Changes in GTH Subunits Transcript Levels in Brain, Pituitary and Ovary at Different Gonadal Stages

**Figure 7:**
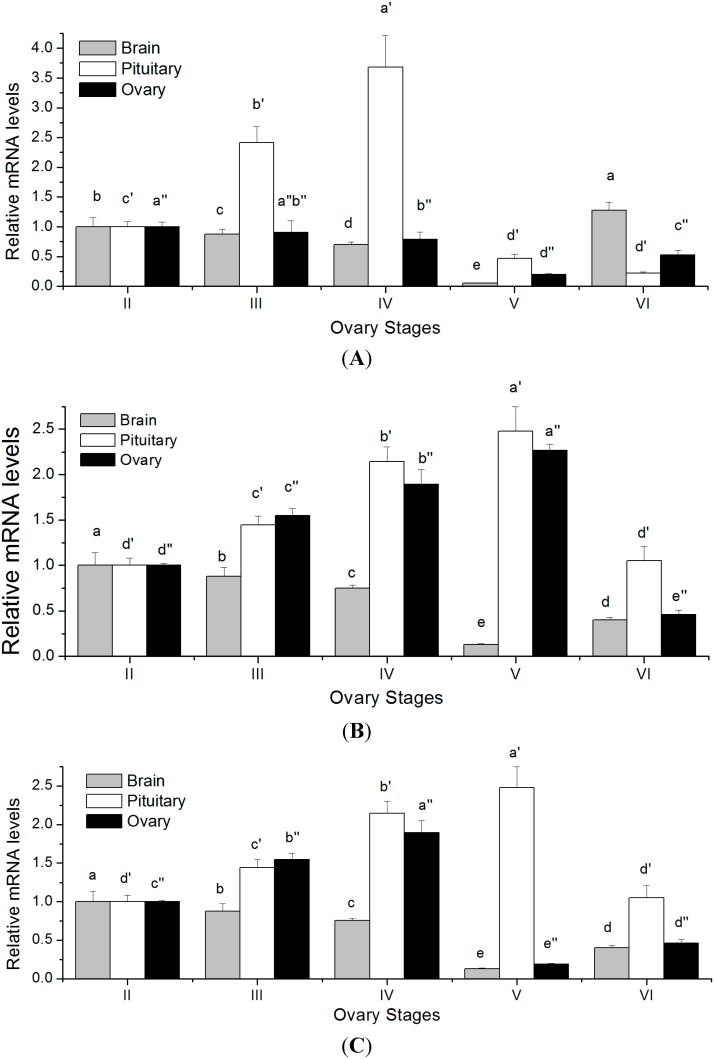
Relative mRNA expression levels of *FSHβ*, *LHβ* and *CGα* mRNA at different ovary stages in maturing *C. semilaevis*. Abundance of *FSHβ* (**A**), *LHβ* (**B**) and *CGα* (**C**) transcripts, respectively, was determined by quantitative real-time PCR and normalized to *18S* (*n* = 4). Each bar represents the mean ± SE. Tissues marked by the different letters differ significantly from each other (*p* < 0.05). The superscript “'” denotes the letter of statistical significance difference in the pituitary at different ovary stages. And the superscript “''” denotes the letter of statistical significance difference in the ovary at different.

The temporal changes of *FSHβ*, *LHβ* and *CGα* mRNA levels in brain-pituitary-ovary axis at different ovarian stages of tongue sole are shown in Figure 7. In pituitary, the transcript levels of *FSHβ* gradually increased with ovarian development. It attained the climax at stage IV, followed by a significant decline during stage V (*p* < 0.05). In brain and ovary, the expression levels of *FSHβ* decreased from stage II to stage V, and reached the lowest values in stage V. From then on, *FSHβ* transcript levels increased significantly in stage VI (Figure 7A). In pituitary and ovary, the transcript levels of *LHβ* were progressively elevated from stage II and reached a maximum in stage V (*p* < 0.05), and then dropped sharply in stage VI. Moreover, the curve for the GSI followed a very similar variation pattern. In brain, the transcript level of *LHβ* fell from stage II to stage V, but significantly increased in stage VI (*p* < 0.05) (Figure 7B).

In pituitary, the transcript level of *CGα* significantly increased from stage II, peaked during stage V, then markedly decreased in stage VI (*p* < 0.05). In brain, the transcript level of *CGα* gradually decreased from stage II to stage V, but increased slightly in stage VI. In ovary, the transcript level of *CGα* increased from low levels during stage II, peaked during stage IV, and significantly dropped during stage V, and then increased during stage VI (Figure 7C).

### 2.6. Plasma Gonadotropins Levels at Different Gonadal Stages

**Figure 8:**
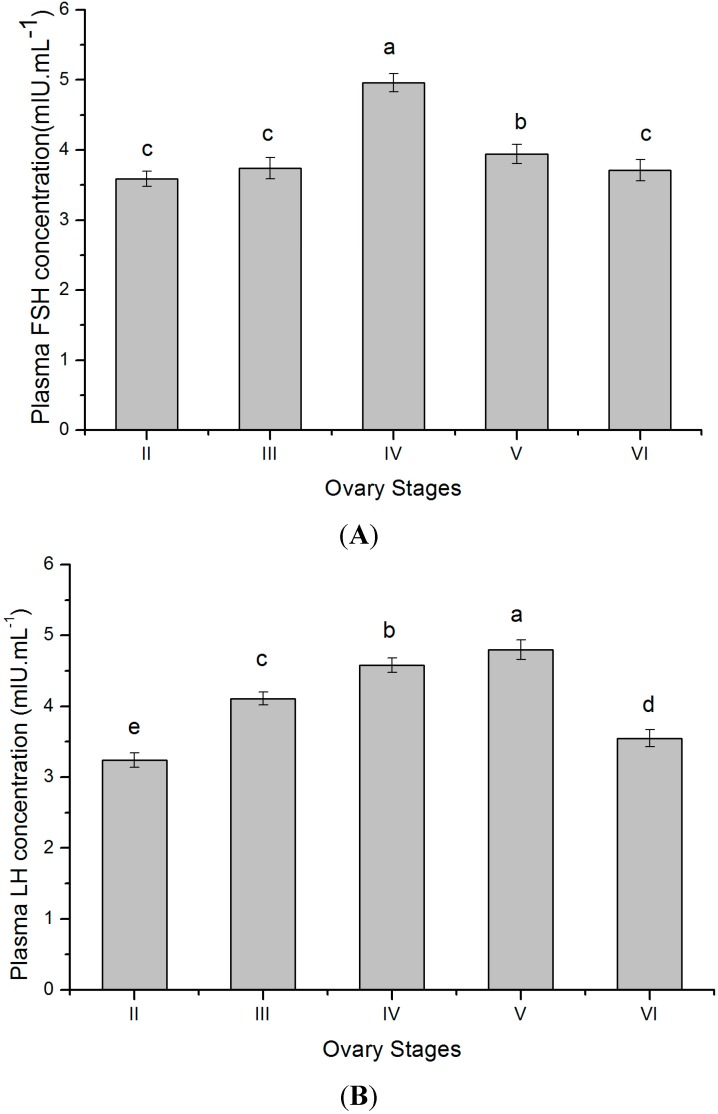
Concentration of plasma FSH (**A**) and LH (**B**) at different ovary stages in maturing *C. semilaevis* (*n* = 4). Each bar represents the mean ± SE. The different letters differ significantly from each other (*p* < 0.05).

Plasma FSH and LH levels of female tongue sole were measured by radioimmunoassay (RIA) to evaluate the relationship between *GTH* subunits transcript level and plasma gonadotropin level. Plasma FSH and LH changes showed a stage dependent variation. In stage II, the high levels of FSH were noted, and the levels showed very high values during stage IV (Figure 8A). Plasma LH levels were relatively high during stage V (Figure 8B). Thereafter, the plasma levels of FSH and LH declined during stage VI. In the present study, the distinct correlation between the plasma hormone levels and the gonadal development stages was observed.

### 2.7. Discussion

In this study, three cDNAs with complete ORF of the tongue sole *FSHβ*, *LHβ* and *CGα* were cloned and characterized, and the spatial and temporal expression of three genes in the brain-pituitary-ovary axis were investigated by qRT-PCR. The plasma levels of GTHs and ovarian histology were examined by RIA and hematoxylin-eosin staining. The analysis of the primary structure of tongue sole FSHβ and LHβ indicated the presence of one *N*-glycosylation site in each polypeptide that is almost conserved among teleosts and higher vertebrates since this site seems to be involved in the biosynthesis and regulation of the hormones [6,32]. In southern catfish (*Silurus meridionalis*), African catfish (*Clarias gariepinus*), channel catfish (*Ictaluris punctatus*), marbled eel (*Anguilla marmorata*) and Japanese eel (*Anguilla japonica*), FSHβ show two putative *N*-glycosylation sites. There was only one N-linked glycosylation site in tongue sole, Senegalese sole and European seabass and red seabream (*Pagrus major*). In orange-spotted grouper and Atlantic halibut (*Hippoglossus hippoglossus*), FSHβ does not have a potential glycosylation site. As suggested in Atlantic halibut [33], the carbohydrate chains of functional importance for tongue sole FSHβ may be also supplied by the CGα.

The tongue sole FSHβ and LHβ both have typically 12 highly conserved cysteines which may form crosslinked disulphide bonds with the CGα, and thus are suggested to be involved in the processes of subunit assembly and receptor binding [34]. However, the tongue sole mature FSHβ lacks the 3rd Cys and shows an additional Cys at the *N* terminus, in agreement with the situation in Salmonids, Perciforms and Pleuronectiformes [6,13,33]. In human, the 3rd cysteine seems to be involved in the formation of the so called “seat-belt” structure that is wrapped around the CGα subunit upon dimerization and appears to be essential for receptor binding and heterodimer formation [35]. So the variation in the primary structure of tongue sole FSHβ may lead to differences in the nature of receptor interactions and the stability of the heterodimer. The tongue sole LHβ mature peptide has some conserved sequences between the 7th and 10th cysteine residue, which have been suggested to be important for heterodimerization, stability and the metabolism of GTHs such as Gly-Val-Asp and Pro-Val-Ala [32,36]. Other key residues suggested for the binding of LHβ to the CGα subunit, such as Cys and Gly in the teleost-specific sequence Cys-Ser-Gly-His between the 4th and 5th cysteine [37], are also shared by tongue sole LHβ.

For tongue sole CGα, 10 cysteines and two putative N-linked glycosylation sites towards the *C* terminus of the mature peptide are conserved. From amino acid sequences comparison, it appeared that one region was highly conserved. This region from 33–66 amino acid residues, consisted of two pairs of adjacent Cys and the first putative N-linked glycosylation site, was believed to be involved in this process of subunit assembly and/or receptor binding in fish [38,39]. The alignment results also showed that fish CGα is more conservative than FSHβ and LHβ. The high conservation of the primary structure of CGα between mammals and teleosts is likely due to the fact that CGα is shared by all pituitary glycoprotein hormones, including FSH, LH and thyrotropin (TSH), and hence it is probably under higher selective pressure during vertebrate evolution.

Comparison of amino acid sequences and phylogenetic analysis of teleost GTHβ subunits indicated that the tongue sole FSHβ showed the highest identities to teleost FSHβ, and that tongue sole LHβ was more similar to teleost LHβ, which provided further structural evidence for the identities of the tongue sole deduced peptides as FSHβ and LHβ. However, phylogenetic analysis showed that neither tongue sole FSHβ nor LHβ clustered with their respective counterparts from Pleuronectiformes fish, suggesting that the primary structure of the GTHβ subunits of this group of teleosts may be highly divergent. One possible explanation for the molecular phylogenic relationships of GTHβ subunits may be that four Pleuronectiformes species come from a different family and genus. Nevertheless, the primary structure of tongue sole LHβ appears to be more conserved than that of FSHβ as has been previously found for other teleosts [40,41,42,43,44]. This fact may indicate a more rapid diversification of fish FSHβ which might be associated with slightly different physiological roles of this hormone among teleosts [40]. The identity of tongue sole CGα was mostly related to those of Pleuronectiformes and Perciformes. The phylogenetic tree of fish CGα showed tongue sole CGα and their respective counterparts from other Pleuronectiformes (*i.e.*, Japanese fiounder and Atlantic halibut) did not form a single cluster, also indicating that the primary structure of CGα of this group of teleosts may be highly divergent.

In multiple spawning fish, the function of GTHs remains largely unknown, despite numerous studies in different species. Currently, there are few methods available for analyzing the function of GTHs in non-salmonids and little understanding of how they regulate gametogenesis in multiple spawning fish. Thus, the gene expression analyses of *GTH* subunits mRNA have predominantly been used to characterize the GTHs function in non-salmonids [16]. In the present study, the highest transcript levels of *GTH* subunits were found as expected in the pituitary. Additionally, three *GTH* subunits mRNA were also detected in the brain, ovary, kidney and liver. However, transcript levels in those tissues were very low. Extrapituitary expression of *GTH* subunits has been previously demonstrated. In gilthead seabream, the mRNA expressions of *FSHβ* and *LHβ* were detected in the oocyte by *in situ* hybridization [19]. In zebrafish (*Danio rerio*), three *GTH* subunits were also found in various tissues, such as brain, kidney, liver, testis and ovary by RT-PCR [44]. In Atlantic cod (*Gadus morhua*), three *GTH* subunits mRNA were detected in ovary by qRT-PCR [45]. In cichlid (*Cichlasoma dimerus*), the distribution of FSHβ and LHβ in the preoptic and hypothalamic areas was found by immunohistochemistry [46]. Based on these data, it was believed that the extrapituitary expression of *GTHs* may be a common phenomenon which was further supported by our results. Some studies were performed to elucidate the physiological implications of extrahypophyseal gonadotropin production [47]. Despite study about the function of GTHs in the brain of fish being scarce, information available from mammals and teleost suggested they may be related to brain development and reproductive behaviors [46,48].

In teleosts, ovarian folliculogenesis is a complex process integrating both systemic endocrine hormones and intraovarian factors. The regulation of oocytes at different developmental stages of multiple spawner might be supported by differential intrafollicular behavior. *In situ* hybridization revealed that *GTHs* transcripts located the cytoplasm of oogonia, previtellogenic and vitellogenic oocytes in the ovary of pejerrey (*Odontesthes bonariensis*). The GTHs could play a role as intraovarian factor during germ cell maturation [49]. Moreover, previous study showed that the *GnRH-III* mRNA of tongue sole was expressed in brain and gonad by qRT-PCR analysis [30]. Therefore, this implied for the ovary of tongue sole a local GnRH-GtH axis may also exist. Taking together these considerations, the functions of GTHs in ovary of tongue sole were accordance with a previous report, in which the GTHs in the gonads of fish were presumed to serve as autocrine/paracrine factors participating in intraovarian communication [19,20].

The synthesis and secretion of GTHs are regulated by positive and negative factors that act at the levels of brain, pituitary and gonad. Also, the temporal differences of *FSHβ*, *LHβ* and *CGα* transcripts were to our knowledge first detected in the brain-pituitary-ovary axis at different ovarian stages of tongue sole. The *FSHβ* transcription in the pituitary gradually increased from stage II to stage IV, which changed in association with an increase of GSI values and plasma levels of FSH. On the other hand, the transcript levels of *FSHβ* in the ovary continuously existed from stage II to stage V. These results were consistent with findings reported for other fish, in which FSH takes part in vitellogenesis [50,51]. Moreover, the circulating levels of plasma FSH in rainbow trout were elevated during vitellogenesis [52]. These reports, together with present data, suggested that this elevation of *FSHβ* transcript levels stimulated the onset of vitellogenesis in tongue sole, whereas the relative low transcript levels of *FSHβ* in both stage V and stage VI may be associated with the ongoing vitellogenesis that runs in parallel with final oocyte maturation and ovulation.

In this study, the *LHβ* transcription in the pituitary and ovary followed the variation of GSI very closely and significantly increased during late vitellogenesis and oocyte maturation compared to early vitellogenesis. A previous study showed an increase in the *LHβ* transcript levels around maturation and spawning in Japanese eel [53]. Furthermore, LH inducing the final maturation of oocytes, as well as the development of the maturational competence of oocytes has already been described in red seabream [54]. On the other hand, it has been suggested that LH may be involved in regulating vitellogenesis in chub mackerel (*Scomber japonicus*) which is a multiple spawning fish, because *LHβ* mRNA levels were high from late vitellogenesis to ovulation [16]. In tongue sole, the high transcript levels of *LHβ* during late vitellogenesis may indicate a role for LH not only in oocyte maturation but also in vitellogenesis in the late phase of ovarian development. Moreover, the present results showed that the pituitary *LHβ* mRNA expression patterns were correlated with circulating LH variation during oogenesis in this species. It has already been reported that in the striped bass (*Morone saxatilis*), plasma levels of LH were low during vitellogenic growth and that a plasma LH surge occurs during final oocyte maturation and ovulation [55], a circulating pattern similar to that found in the present work. Furthermore, histological analysis of the gonads reflected the fact that the ovary of tongue sole contains clutches of oocytes at several stages of development, and therefore there is need for the action of both FSH and LH for continuous ovarian maturation and ovulation throughout the spawning season. Similar expression patterns of pituitary *FSHβ* and *LHβ* genes are found in goldfish (*Carassius auratus*), blue gourami (*Trichogaster trichopterus*) and three-spined stickleback (*Gasterosteus aculeatus*), which are also multiple spawners with oocytes showing asynchronous development [11,12,15,56]. In Senegalese sole, the levels of *FSHβ* and *LHβ* transcripts in the pituitary of males increased during winter and spring, at the time when testicular germ cell development and spermatozoa production were stimulated [57]. These results suggested that FSH and LH may regulate spermatogenesis of Senegalese sole, which was similarly to that described for other teleosts with synchronous germ cell development. So our finding that both *FSHβ* and *LHβ* are expressed at the different gonadal stages is in accordance with the multiple spawner nature of tongue sole.

The changes of *CGα* transcription in pituitary were similar to those for *LHβ* during the reproductive cycle, peaking at the stage of oocyte maturation. The increase in the transcript levels of *CGα* may be the result of a rise in β*-*subunits transcription at the different ovarian stages of tongue sole. Therefore, such parallelism may indicate that these genes share similar regulatory elements, which is not surprising given the common ancestral origin of three subunits [58]. In addition, the brain *GTHs* genes were expressed at the different ovarian stages, although the transcript levels gradually decreased along with ovarian development in this flatfish. Recently, immunoreactive neurons in the preoptic area of cichlid were found to send projections through different brain areas and neurohypophysis. An individual pituitary *in vitro* culture system further revealed that the both pituitary gonadotropins were regulated by brain-derived gonadotropins [46]. Thus, such regulatory axes may contribute to or finely tune neuroendocrinology of reproduction in tongue sole. However, research in this direction has yet to be conducted.

Since the late 1980s, GTHs have been isolated and characterized in fish, and GTHs homologous immunoassays have been developed for limited species with synchronous ovarian development [59,60,61,62,63,64]. The GTHs immunoassay for multiple spawning fish have only been developed for Nile tilapia and mummichog (*Fundulus heteroclitus*) [17,65]. However, tilapia is a tropical species without a distinct reproductive cycle. In fact, the spawning season variations in plasma GTHs levels of mummichog were first reported in multiple-spawning fish. In tongue sole, the FSH and LH have not yet been purified, thus it is not possible at the moment to develop a specific immunoassay to determine the plasma levels of the important hormones during oogenesis. Currently, the plasma circulating levels of FSH and LH have been examined by the heterogenous antibody. When compared with the well-known rainbow trout pattern, the remarkable difference is in the FSH profiles, especially the high level in the maturation phase. In rainbow trout, plasma FSH levels significantly increased at the onset of vitellogenesis, were maintained during vitellogenesis and then decreased prior to maturation, while plasma LH levels peaked at maturation–ovulation [52,66]. The present results indicated an active synthesis and secretion of FSH during the spawning season. Evidently, the high FSH levels would be necessary for the successive spawning, resulting from the successive production and development of gametes. In tongue sole, the high levels of LH were observed during the maturation phase, and the levels in the ovulation of the spawning period slightly decreased. Thus, LH is the important hormone that is responsible for the final maturation of oocytes in tongue sole. However the relatively high levels of LH during the vitellogenic phase concomitant with the surge of FSH suggested that in tongue sole, LH may play a role not only during final oocyte maturation, but also during vitellogenesis.

It must be noted that plasma peptide hormone levels are controlled not only by the transcription, but affected thereafter by translation, processing and accumulation, exocytosis, blood clearance, *etc.* Alteration in the level of *GTH*s gene expression is not necessarily a reliable yardstick for the plasma level of the hormones [6]. Taken together, these results indicated the importance of FSH and LH for various reproductive events in this flatfish. Nonetheless, the precise information as to the physiological role of FSH and LH remains to be determined. So it would unsettle us to have to infer their specific roles in oocyte growth, in stimulating the synthesis of various steroids, and in stimulating the acquirement of the oocyte maturational competence, oocyte maturation and ovulation.

## 3. Experimental Section

### 3.1. Experimental Fish and Sample Collection

The adult female tongue soles (body weight, 1266.3–2271.0 g; body length, 53.0–66.0 cm) were reared in a fish farm of Rizhao, Shandong China. Before the start of experimental sampling, they were reared in natural sea water under controlled conditions (10–25 °C; ≥5 mg/L O_2_; 7.8–8.4 pH). At the various gonadal developmental stages, female fish (*n* = 4) were decapitated after anesthesia and a record made of weight and length. The gonads were excised and measured, and then the gonadosomatic index (GSI = [Gonad weight/(Body weight − Viscera weight)] × 100) was calculated. The midsection of each ovary from an individual was taken and fixed in Bouin’s solution for hematoxylin and eosin (H&E) staining in order to identify the developmental stages of the ovary. The brains were removed following anesthetization, snap-frozen in liquid nitrogen, and stored at −80 °C until total RNA extraction. The pituitaries were carefully detached from the brains tissue, snap-frozen in liquid nitrogen and stored at −80 °C before proceeding to RNA extraction. Ovaries were dissected and one portion was snap-frozen in liquid nitrogen. Similarly, different tissues and organs of female tongue sole were removed, immediately frozen in liquid nitrogen and finally stored at −80 °C, until later use for total RNA extraction. The treatment of fish adhered to the guidelines of animal experiments set by the Yellow Sea Fisheries Research Institute.

### 3.2. Histological Analysis

Fixed ovary segments were dehydrated in a 70%–100% ethanol series, cleaned in xylene, embedded in paraffin wax, and 5 μm sections were performed by microtome (LEICA-RM2235, Wetzlar, Germany) and stained with haematoxylin (Sigma-Aldrich, St. Louis, MO, USA), counter stained with eosin (Sigma-Aldrich), and then photographed by light microscope (LEICA DM500, Wetzlar, Germany). Five phases of ovarian development were identified accord to the method applied to the Senegalese sole [67]: Previtellogenesis (stage II), vitellogenesis (stage III), late vitellogenesis (stage IV), maturation (stage V), after ovulation (stage VI).

### 3.3. Total RNA Extraction and Reverse Transcription (RT)

Samples stored at −80 °C were immediately transferred to cold RNAiso reagent (Takara, Dalian, China) to preserve RNA quality. Total RNA was extracted following the manufacturer’s protocols. The quality of RNA was measured at A260 nm/A280 nm (Nanodrop, Thermo Scientific, Wilmington, DE, USA). Only RNAs with A260 nm/A280 nm ratios of 1.6–2.0 were used. RNA concentration of each sample was determined, and an agarose gel was applied to check RNA integrity. Then, the first-strand cDNA for isolation of three GTH subunits cDNA fragments was synthesized with 1 μg total RNA of pituitary gland by using PrimeScript 1st Strand cDNA Synthesis Kit (Takara, Dalian, China) in a 10 μL reaction.

### 3.4. Isolation and PCR Amplification of FSHβ, LHβ and CGα cDNA Fragments

In order to clone *GTH* subunits fragment of the tongue sole, three pairs of degenerate primers (FSHβ F/R for *FSHβ*, LHβ F/R for *LHβ* and CGα F/R for *CGα*) , listed in Table 1, were designed. PCR reaction was carried out in a final volume of 25 μL containing 1 μL of cDNA from pituitary tissue, 2.5 μL of 10× PCR buffer, 2 μL of a 2.5 mM dNTP mix, 0.5 μL of each primer, 0.2 μL of Taq polymerase (Takara, Dalian, China). Thermal cycling comprised 94 °C for 5 min followed by 30 cycles 94 °C for 30 s, annealing at 60.8 °C (*FSHβ*), 58.2 °C (*LHβ*) and 55.1 °C (*CGα*) for 30 s, and an extension temperature of 72 °C for 30 s, followed by a final extension of 72 °C for 10 min. All PCRs were performed in a thermal cycler (Bio-Rad S1000, Hercules, CA, USA). The PCR products were separated through 2% agarose gel and purified using the E.Z.N.A Gel Extraction Kit (Omega Bio-Tek, Winooski, VT, USA) and then cloned into the *pEASY*-T1 Cloning Vector (TransGene Biotech, Beijing, China), and three clones of each gene were sequenced using the ABI3730XL sequencer (ABI, Foster City, CA, USA).

**Table 1:** The primer sequences used in the present study.

Name	Sequence (5'→3')	Usage	Size (bp)
FSHβF	AGCAGAGGATGMAGCTGGT	Degenerate primer	241
FSHβR	TGGCCACAGGGTAGGTSAC		
FSHβGSP1	GCCACAGGGTAGGTCACTTCCTCTGGAC	5' RACE primer	321
FSHβNGSP1	GGTCCAGTCTCCGTTGCACGTCTTCTGC	Nested 5' RACE primer	268
FSHβGSP2	GTGCTGGCGATGGTTTGTCCTGGGAAGG	3' RACE primer	409
FSHβF1	TGATGGGTGTCCAGAGGAAG	qRT-PCR primer	95
FSHβR1	CAACAAACCGTCCACAGTCC		
LHβF	CCATYTGCAGCGGYCAC	Degenerate primer	162
LHβR	GCAGCTCAMAGCCACMGG		
LHβGSP1	GCTCAAAGCCACAGGGTACGTGATGGTG	5' RACE primer	399
LHβGSP2	AAGATGCCGTCGTTTCAGTCACCCTTCA	3' RACE primer	353
LHβF1	AGACGGTGTCTCTGGAGAAAGAAG	qRT-PCR primer	105
LHβR1	ACGGCACCTTGATGTTTGGT		
CGαF	TAGYTGATTCTTACCCCARCAT	Degenerate primer	270
CGαR	TGCAGTGRCAGTCTGTGTGGTT		
CGαGSP1	CACCACAATACCAGCCACCACTACCTCA	5' RACE primer	340
CGαNGSP1	GCACGTCGCCTCTGAAGTGATGTTCTTT	Nested 5' RACE primer	292
CGαGSP2	GTCTACCAGTGCCAGGGCTGCTGCTTCT	3' RACE primer	441
CGαF1	TTCCCCACTCCTCTAACGACA	qRT-PCR primer	116
CGαR1	ACCACAATACCAGCCACCACTAC		
18S F	GGTCTGTGATGCCCTTAGATGTC	Internal control	113
18S R	AGTGGGGTTCAGCGGGTTAC		

### 3.5. Rapid Amplification of cDNA 3' and 5' Ends (3' and 5' RACE)

SMART™ RACE cDNA Amplification Kit (Clontech, Mountain View, CA, USA) was used for 5' and 3' ends RACE-PCR. To isolate 3' ends of *FSHβ*, *LHβ* and *CGα* sequences, three gene specific antisenses, FSHβGSP2 (for *FSHβ*), LHβGSP2 (for *LHβ*), CGαGSP2 (for *CGα*), were designed and used in 3'-RACE cDNA amplification system. The 5' end of cDNAs was amplified by two rounds of PCR. Primers FSHβNGSP1 (for *FSHβ*), LHβNGSP1 (for *LHβ*) and CGαNGSP1 (for *CGα*), were prepared for touchdown PCR as described by manufacturer’s instructions. Both 5' and 3' RACE PCRs were performed in 50 μL using Clontech Advantage 2 Polymerase Mix according to the manufacturer’s protocol. The PCR products which had the corresponding predicted length were excised, purified and cloned into vector, then sequenced as described above. The list of primers used is presented in Table 1. BLASTN searches were used to verify gene identity and determine similarities with other vertebrates.

### 3.6. Sequence Analysis and Phylogenetic Analysis

Deduced amino acid sequences of the *FSHβ*, *LHβ* and *CGα* cDNAs were analyzed *in silico* with the SignalP 3.0. and NetNGlyc 1.0 programs (http://www.cbs.dtu.dk/services/NetNGlyc), to predict signal peptide sequences and putative N-linked glycosylation sites, respectively. Multiple protein sequence alignment was performed using the CLUSTAL_X program. MEGA 4.0 software package was used to construct and analyze phylogenetic tree using the Neighbor-Joining method with 1000 bootstrap trials. Protein sequences used for analysis and their GenBank Accession Nos. are as follows: *Cynoglossus semilaevis* (AFC90009, AFF59207, AFD04550), *Solea senegalensis* (ABW81403, ABW81404, ABW81405), *Hippoglossus hippoglossus* (CAD10501, CAD10502, CAD10503), *Paralichthys olivaceus* (AAK58601, BAB47388, AAK58600), *Dicentrarchus labrax* (AAN40506, AAN40507, AAK49431), *Epinephelus coioides* (AAO31971, AAM28896, AAN18038 ), *Channa maculate* (AAS01610, AAS01609), *Trichogaster trichopterus* (Q9PW99), *Morone saxatilis* (Q91120, AAB66489), *Pagrus major* (BAB18563, BAB18564, BAB18562), *Oreochromis mossambicus* (AAK83079), *Oreochromis niloticus* (AAP49576, AAP49577), *Acanthopagrus schlegelii* (ABQ96863), *Takifugu niphobles* (BAJ12081), *Monopterus albus* (AAN77069), *Odontesthes bonariensis* (AAP85606, AAP85607, ABD36561), *Fundulus heteroclitus* (P30971, P47744), *Danio rerio* (NP_991187, AAV31153, AAR84285), *Carassius auratus* (Q98848, Q98849, AAV65764), *Cyprinus carpio* (O13050, P01235), *Ictalurus punctatus* (Q9DG81, Q9DG80, AAD18004), *Clarias gariepinus* (P53543, P53542), *Silurus meridionalis* (AAY42268), *Oncorhynchus mykiss* (BAB17686, BAB17687, BAB17685), *Oncorhynchus masou* (P48252, P48253), *Brachymystax lenok* (AAR99810, AAR99811), *Coregonus autumnalis* (P48250), *Plecoglossus altivelis* (AAM92269, AAM92270), *Oncorhynchus keta* (P10256), *Anguilla Anguilla* (AAN73407, P27767, P27794), *Anguilla japonica* (Q9YGK3, BAD14302), *Conger myriaster* (BAB97390, BAB97391), *Acipenser baerii* (CAB93504, CAB93502, CAC43060), *Acipenser gueldenstaedtii* (AAS92716), *Gasterosteus aculeatus* (CAD59185), *Gadus morhua* (ABD62882), *Sebastes schlegelii* (AAU14140), *Rana japonica* (BAD16757), *Mauremys reevesii* (BAB92948), *Coturnix japonica* (BAC01164), *Homo sapiens* (NP_001018090, NP_000885, NP_000726).

### 3.7. Quantitative Real-Time PCR

To examine the *FSHβ*, *LHβ* and *CGα* transcript levels at the different tissues of the sexual maturation tongue sole, quantitative real-time PCR (qRT-PCR) was conducted on a Mastercycler ep *relplex* (Eppendorf, Hamburg, Germany). Further, the qRT-PCR was used to study the changes of *GTH* subunits mRNA transcript levels in the whole brain, pituitaries, and ovaries at the different ovarian stages. The qRT-PCR was performed using the primers FSHβF1/R1 for *FSHβ*, the primers LHβF1/R1 for *LHβ*, and the primers CGαF1/R1 for *CGα* (Table 1). *18s* rRNA was used for normalization of the expression levels. Total RNA extraction and quality measure were processed as described in Section 3.3. RNA samples were reverse transcribed using PrimeScript RT reagent Kit With gDNA Eraser (Perfect Real time) (Takara, Dalian, China) following the manufacturer’s instructions. The gDNA Erase in this Kit can eliminate genomic DNA contamination. Amplifications were carried out in a final volume of 20 μL, containing 10 μL SYBR Premix Ex Taq (2×) (Takara, Dalian, China), 0.8 μL of each forward and reverse primer, 1 μL cDNA and 7.6 μL PCR-grade water. The reaction carried out without using the template was used as blank control. The PCR amplification procedure was initial denaturation at 95 °C for 30 s, 40 cycles of 95 °C for 5 s and 60 °C for 20 s, followed by disassociation curve analysis to determine target specificity. For normalization of cDNA loading, all samples were run in parallel using the housekeeping gene as loading standard for tissues and brain-pituitary-ovary at the different ovarian stages, respectively. To estimate efficiencies, a standard curve was generated for each primer pair based on known quantities of cDNA (10-fold serial dilutions corresponding to cDNA transcribed from 100 to 0.01 ng of total RNA). All calibration curves exhibited correlation coefficiencies and were in the range 0.91–0.98. Relative mRNA expression was determined using the 2^−ΔΔ*C*t^ method [68]. Agarose gel electrophoresis of the PCR products was performed to confirm the presence of single amplicons of the correct predicted base-pair sizes.

### 3.8. Radioimmunoassay

The tongue soles were anesthetized with 3-aminobenzoic acid ethyl ester (MS222, Sigma, St. Louis, MO, USA). The blood was collected from the caudal vasculature by heparinized disposable syringes. Blood samples were put in ice-cold heparinised tubes and held on ice before centrifugation at 12,000× *g* for 10 min at 4 °C. The plasma was aliquoted into 1.5-mL plastic microfuge tubes and stored at −40 °C until analysis. Serum levels of FSH and LH were measured using commercial kits from Diagnostic Products Corporation (Tianjin Nine Tripods Medical & Bioengineering Co., Ltd., Tianjin, China). Hormone levels were determined by radioimmunoassay (RIA) according to the manufacturer’s instructions [69,70]. The assay detection limits were 1.0 mIU·mL^−1^ for FSH and 0.9 mIU·mL^−1^ for LH, respectively. The inter- and intra-assay coefficients of variation for FSH and LH were 5.4%–5.5% and 7.5%–8.7%, respectively. The cross-reactivities of antibody for FSH to TSH, LH, HCG and PRL were 3.0% × 10^−3^, 1.1% × 10^−2^, 1.6% × 10^−2^ and 1.4% × 10^−3^, respectively. The cross-reactivities of antibody for LH to TSH, FSH, HCG and PRL were 2.0% × 10^−3^, 2.7% × 10^−2^, 5.3% × 10^−1^ and 1.3% × 10^−3^, respectively.

### 3.9. Statistical Analysis

Statistical analyses were performed with SPSS 13.0 software (SPSS Inc., Chicago, IL, USA). All assays were performed in triplicate. All data were expressed as mean ± SE and analyzed by one-way ANOVA followed by Duncan’s multiple comparison tests. Statistical significance was considered as *p* value of <0.05.

## 4. Conclusions

In summary, three cDNAs encoding the *CGα*, *FSHβ* and *LHβ* subunits were first isolated and characterized in half-smooth tongue sole. The qRT-PCR and RIA analysis first suggested that GTH subunits played an important role through the brain-pituitary-ovary axis endocrine system in this flatfish. In addition, the recombinant GTHs, in combination with pharmacological studies of the tongue sole GTH receptors and their precise cellular localization, in the future, will hopefully help to understand the specific physiological function of GTH subunits to enable the design of novel methods to overcome reproductive dysfunctions of the cultured female tongue sole.
